# Environmental and capacity requirements are critical for implementing and sustaining a drug prevention program: a multiple case study of “Clubs against drugs”

**DOI:** 10.1186/1747-597X-9-6

**Published:** 2014-01-25

**Authors:** Elin Norrgård, Ewa Wikström, Chris Pickering, Johanna Gripenberg, Fredrik Spak

**Affiliations:** 1 Department of Public Health and Community Medicine, Unit of Social Medicine, University of Gothenburg, Institute of Medicine, 40530 Gothenburg, Sweden; 2 Section of Management and Organisation, University of Gothenburg, School of Business, Economics and Law, Gothenburg, Sweden; 3 Department of Psychiatry and Neurochemistry, Addiction Biology Unit, University of Gothenburg, Institute of Neuroscience and Physiology, Gothenburg, Sweden; 4 Department of Clinical Neuroscience, Stockholm Centre for Psychiatric Research and Education, Karolinska Institutet, Solna, Sweden

**Keywords:** Community based prevention, Community capacity, Implementation, “Club drugs”, Prevention

## Abstract

**Background:**

“Clubs against drugs” (CAD) is a comprehensive program with a systems approach to prevention with the intention of preventing drug use in nightclub environment. In 2001 CAD was developed and implemented in Stockholm and was disseminated to 20 other municipalities in Sweden up until 2010. This study investigates the factors related to the implementation and compares Stockholm to the rest of the municipalities.

**Methods:**

A sequential exploratory method was used which consisted of three steps including a questionnaire and two rounds of interviews. Questionnaires included all communities and the interviews included twelve respondents from three example municipalities in the first phase and four respondents from Stockholm in the second phase. The interviews were analyzed using content analysis.

**Results:**

In Stockholm the program was described as having been implemented and sustained over time. The implementation in the example municipalities was perceived as difficult with many obstacles and achieving sustainability was also described as difficult. Two of three municipalities were not active at the time of this study, illustrating that the program only lasted a few years. Factors identified as being related to implementation outcomes were need assessments, participation, support, collaboration and local enthusiasts.

**Conclusions:**

The capacity to implement and sustain CAD differed substantially between Stockholm and the other municipalities. If the prerequisites and capacity are not sufficient the implementation is not likely to be successful, even when the activities are promoted on a national level like CAD. The needs of the interventions and the capacity to implement the program should be examined before adopting the program. This was not done, probably because the drive to spread the activity was not sufficiently questioned.

## Background

This study investigated implementation of the “Club Against Drugs” (CAD) program [1] in 20 municipalities throughout Sweden. The CAD program was developed in Stockholm in 2001 and dissemination to the rest of the country began in 2002. The decision to adopt a program does not automatically lead to its successful implementation. Implementation should rather be regarded as a time-consuming process, then as an action [2]. Numerous and complex factors influence program implementation and this complexity has meant that many initiated implementations are unsuccessful. Implementation always includes change and is dependent on many factors including needs, resources, leadership and capacity. Further, a new program has to have a clear advantage over current practice [2]. Part of the process is also sustainability to ensure that the program survives beyond the initial implementation phase, thus enabling beneficial long-term outcomes [3,4]. To facilitate implementation and sustainability of a program sufficient capacity within the community and within the organizations involved is required [2,5]. A common discrepancy exists between the requirements for implementing research-driven prevention programs and the actual capacity of the communities which are going to use them [6]. This capacity should be examined before the implementation is initiated and considered in the planning phase to avoid a waste of resources [7,8]. The concept of community capacity is complex, evolving and has numerous definitions focusing on varied factors [9], and can be viewed as both an outcome of community efforts or as a prerequisite for implementation [10]. Capacity, broadly defined, includes the resources of the community, organizations and individuals which can be used to accomplish their goals and can include knowledge, resources, collaboration and leadership [9]. Community capacity is in this study defined as the capacity of the nightclub stakeholder community as a whole to implement and sustain the comprehensive preventive program CAD. This capacity is based on the ability and resources of the participating organizations and individuals. In the work with CAD a major component was the creation of community coalitions [10] in order to bring together the different capacities.

Many issues and problems arise with the implementation of community-based drug prevention programs because they are complex and do not fall within the framework of one single organization’s boundaries. Instead they require the efforts of many different organisations. Collaboration has thus become an essential element in the success of community-based interventions as well as the reliance on community coalitions to solve complex problems [11]. Collaborative activities between organisations can be difficult to position within the normal organisational structure and a problem with creating collaboration as a separate organisational form is the difficulties integrating the collaborative experiences into the permanent organization [12]. The difficulties with coalitions will arise if the potential benefits may not materialize in reality. Coalitions demand resources and commitment and the problems coalitions set out to solve are complex and hard to change [10].

The CAD-program was developed in 2001 by researchers in Stockholm and focused on reducing the availability and use of illegal drugs in the nightclub environment. The program was developed, implemented and managed by STAD (Stockholm prevents Alcohol and Drug problems), a research and development unit within the Stockholm County Council and the Karolinska Institutet. Initially, a study was performed to assess the need for a community-based drug intervention and to gain ideas from community stakeholders on how such a program should be designed. The results indicated that staff at licensed premises had a higher prevalence of last year drug use than the general Swedish population and that observed drug use among guests was high [13]. The program was theoretically based on a systems approach to prevention [14] and as there were no relevant studies examining prevention of drugs in licensed premises the researchers relied on existing research from the alcohol field. The main objective of the program was to decrease availability of club drugs at licenced premises but also to increase knowledge among bar staff and in the community as a whole. The methods used were drug training for doormen and other staff at licenced premises, policy work, increased enforcement, environmental changes and media advocacy. Researchers reported that the community readiness was high and that there was an extensive support for the program [1]. CAD did have an effect on the behaviour of doormen and led to a higher degree of intervention towards apparently drug intoxicated guests [15,16].

In 2002 the Swedish authorities responsible for drug prevention decided to disseminate CAD to other municipalities in Sweden. As a result Gothenburg and Malmö, the second and third largest cities in Sweden, adopted the program and by 2010 a total of 20 communities besides Stockholm had initiated the program. The program was to some extent changed from how it had originally been developed and implemented in Stockholm; e.g. the required length of the education for bar staff was reduced from 2 days to half a day. The core components of the program were *collaboration*, *education* and *inspection*.

For the municipalities and clubs that initiated the program there were a number of demands on their performance and level of activity. Mandatory components for the municipalities included political anchoring, formation of a steering group, conducting a survey examining the situation in clubs and offering education in collaboration with the police. Recommended components included assigning a local coordinator, performing a study using actors (like the one in Stockholm) and offering policy education to club managers. Mandatory components for the clubs were making environmental rounds with a representative from the municipalities’ steering group, working actively with policy, cooperating with the police and educating the staff or at least the key persons. For more details see the CAD website krogarmotknark.se. There were no formal demands for the police. Some funding was available from the Swedish National Institute of Public Health and the additional funding was taken from the general operating budgets of the stakeholders. A national steering group was set up and was responsible for managing the contact with the communities, updating the website and arranging national conferences. More municipalities wished to join but had to wait for the results from an evaluation. As this was taking longer than planned more municipalities were allowed to start working with the program from 2011 and forward.

This study investigates the implementation of CAD in the municipalities to which it has been disseminated in contrast to the findings from Stockholm. We wanted to identify what central factors facilitate and hinder implementation and sustainability of a comprehensive prevention program involving multiple stakeholders. We relate these factors to the concept of community capacity and argue that without sufficient capacity one should question the initiation of an implementation.

## Methods

The “Clubs against drugs” program was developed in Stockholm and had in 2010 been disseminated to 20 other municipalities in Sweden. A sequential explanatory mixed method [17] was used for this study and consisted of three stages that began with a questionnaire followed by two rounds of interviews. All municipalities were included in the first step which consisted of a web-based questionnaire that was sent out 2012. Using information from the local CAD coordinators, the questionnaire was sent out to all participants in the CAD program in that municipality. Data from the 20 municipalities to which CAD had been disseminated were in the first step analyzed. The answers to these questionnaires were then used to produce questions for the interviews and to assist in the selection of three example municipalities. These were chosen for further investigation using a structural sampling based on size, location and outcome (good, poor or neither). However, it was difficult to select example communities on the basis of outcome as we did not consider implementation in any community outside of Stockholm as particularly successful.

Interviews were conducted with key informants that included public officials, politicians, police, club owners and doormen from each of the three selected municipalities. Unfortunately, doormen were not interviewed as none were willing to participate. The informants were selected with help from the local CAD coordinators. Twelve people, four from each municipality, were interviewed during the summer and autumn of 2012.

Based on the content analysis of these twelve interviews, we wanted to compare the results to those of Stockholm. To do this we interviewed participants from the Stockholm CAD program and asked questions particularly related to implementation factors. The four key informants were the researcher who had been a part of the team at STAD that developed and implemented the initial work, the current coordinator, a police officer who had been involved in the initiation of CAD and a club owner who had also been involved from the start of the program. These interviews were conducted spring 2013. The interviews were semi-structured and conducted by a researcher with a public health background. The first interviews were performed face-to-face and the location was chosen by the respondent and in all cases but one it was held at the respondent’s work place. The Stockholm interviews were performed over the phone. The key questions included how the CAD program was initiated and how the implementation process had progressed, what factors had enabled the process and had there been any barriers, how the collaboration had functioned and what they thought about the future and the sustainability of CAD. The questions asked in the interviews were open ended and gave the respondents space to formulate their answers quite freely. The interview respondents were informed that they would be anonymous and that they could refuse to answer questions or terminate the interview at any point. They were further informed that the interviews would be recorded and were given the opportunity to decline to this.

The interviews were transcribed and analyzed using content analysis techniques [18]. The material was first read through several times. The text was then divided into meaning units, that is constellations of words or sentences that relate to the same central meaning. These were then condensed into smaller units, changing the words but keeping the essence and meaning. Then these were divided into categories and grouped into central themes. A second researcher also reviewed these classifications in order to verify the accuracy of the content analysis.

We did not require ethical approval for this study because it did not handle sensitive information about individuals (Swedish Law (2003:460, 3 § and 4 §) about ethical approval of research that pertains to humans).

## Results

### Questionnaires

A total of 55 surveys were returned, out of 101 distributed, giving a response rate of 54%. Fourteen surveys were excluded because the respondents were not involved in CAD during 2008-2010, thus a total of 41 surveys were included in the analysis. Survey respondents were directly involved in the implementation of CAD and included public officials, politicians, club owners, bar staff, doormen and police officers. Response rate was highest among coordinators and roughly equal for politicians, police and club owners. Response rate was lowest among bar staff and doormen.

The majority of respondents stated to have strengthened relations and knowledge and increased their collaboration through CAD participants; a smaller part also obtained a better knowledge of methods and increased understanding of other’s work role (Table 1).

**Table 1:** What participants received as a result of CAD participation

**Outcome**	**% (n)**
New or strengthened relations	63 (26)
Deeper knowledge in the area	76 (31)
Increased collaboration	76 (31)
Method knowledge	39 (16)
Better understanding of other’s work role	37 (15)

Perceived support differed between the police officers and the rest of the respondents (Table 2). Roughly half of the police officers felt they had sufficient time, finances and support from employers/managers to work with the CAD program. However, approximately 75% of those from the remaining job categories combined found this support sufficient, suggesting a difference in priority between stakeholders.

**Table 2:** How respondents perceived supporting factors in relation to their work with CAD

	**Police officers (n = 8)**		**All but Police officers (n = 33)**	
Type of support	% Perceived as sufficient (n)	% Perceived as insufficient (n)	% Perceived as sufficient (n)	% Perceived as insufficient (n)
Financial resources	63 (5)	25 (2)	77 (24)	21 (7)
Support from employers/managers	50 (4)	50 (4)	88 (29)	9 (3)
Time	50 (4)	50 (4)	67 (23)	24 (8)

Numbers do not add up to 100% as some answered “Don’t know”.

Of the police officers, bar staff and club owners 87% had participated in a CAD educational training and 86% felt that this training had been sufficient for them to feel secure in their roles. All the club owners answering the questionnaire (n = 6) stated that they had developed a drug policy for their club during CAD and four of these perceived the support for this had been adequate. Out of all stakeholders excluding the police 81% answered that the collaboration with the police had been improved. Of the public officials 58% stated that they had completed a survey measuring the negative effects of narcotics. 83% of the respondents answered yes to the question of whether they would participate in CAD if asked today.

### Interviews with respondents from the three example municipalities

The categories and themes from the interviews conducted in the three example municipalities are shown in Table 3. The results connected to each theme are presented below.

**Table 3:** Categories and themes that emerged from the interviews

**Category**	**Theme**
CAD is important	
Support from politicians and employers	Conditions for the implementation
Resources	
National network	
Activities within CAD	
Working together	The implementation
Relying on dedicated individuals	
Lessons learned	Sustainability and development
This must be continual work	

#### Conditions for the implementation

The respondents were very interested in working with drug prevention and believed that the “Clubs against drugs” program was an appropriate way to deal with the problem of drug use in the nightclub environment.

*We were asked by the municipality if we were interested in working with this. Of course we wanted to. It says in the law that this is something that you shouldn’t do, drugs and so on. For the club there was not any doubt, it’s a highly current issue.* Club owner

The support from local politicians and employers was emphasized as an important factor for implementing CAD. The perceived support differed between individuals and municipalities and many perceived this as lacking.

*Interest exists sporadically, but when the employers do not have this interest and it comes as a directive that “This is a problem and we shall do something about it”, it does not get done. Sorry.* Police officer

The respondents, in both interviews and questionnaires, expressed that sufficient resources were necessary to complete program implementation. Resources coming from external sources were perceived as being sufficient but many respondents described the police resources as insufficient.

#### The implementation

The activities completed in the municipalities varied. In the three example municipalities the local coordinator for CAD initiated the use of the program. In all municipalities a steering group comprised of key personnel was created to support and lead the local work. At the time of the interviews club environment rounds had partly been done in two of the example municipalities and in the third this work had not yet been initiated. In two of the example municipalities the work with CAD had stopped almost completely and they did not, at the time of study, have any activities running.

Collaboration was, for all of the interviewees, viewed as a key aspect of local and national work with CAD. Failed collaboration was perceived as a leading cause in the communities where the work had not gone as planned and successful collaboration was described as an essential factor in the community where the work was continued.

The club owners and the police officers thought it was important to have a closer relationship with each other. Police officers described the relationship as positive due to the large number of criminal persons that frequently visited these kinds of venues and cited the advantage of becoming more familiar with the club area during the daytime hours.

*You work with honesty and it is not my fault if someone comes into the club with drugs but rather something we should stop together. So the collaboration was absolutely the best and working with this was best if you viewed it locally, for us.* Club owner

The other stakeholders considered the police to be the weakest link.

*If we look at this collaboration as resting on three legs – police, club owners and municipalities, the weakest leg in this triad is the police.* Public official

The reasons mentioned for the difficulties collaborating with the police were related to opinions of organizational weaknesses within the police resulting from many reorganizations and also transfers and lack of interest from the police authorities regarding preventive work. This was also the opinion expressed by the interviewed police officers. One of them said that their work in large was determined by acute actions rather than a long term prevention perspective.

The police were described as an important part of the CAD work and the other stakeholders did not think it was possible to implement the program without their active participation. If one or a few of the club owners were not interested the work could still continue. According to the respondents the municipalities were important but their role was not as critical compared to that of the police.

*It doesn’t help if we identify that someone is under influence of drugs if there are no police that can handle it. It’s not the job of the doormen; they have some authority but cannot act as a police. That is what the police are supposed to do, but the police don’t exist.* Club owner

According to the respondents the work in many municipalities depended on a few dedicated individuals who were pushing the work forward.

As long as there is not a developed organization dedicated individuals are needed to keep it going.

Police officer

#### Sustainability and development

The CAD program was described as a long-term commitment and in need of a plan for sustainability. The respondents talked about lessons they had learned from CAD. One police officer thought that the police had been too unrealistic concerning what they would be able to contribute with in the program and not been able to live up to their promise.

*One can be wise in hindsight and say that we should have obtained the resources that were needed*. Police officer

Formal agreements were suggested as a possible solution to this.

### The interviews from Stockholm

The categories and themes from the interviews conducted in Stockholm are presented in Table 4. The results connected to each theme are presented below.

**Table 4:** Categories and themes from the interviews in Stockholm

**Category**	**Theme**
The needs of the community	
Resources	
Support from managers and politicians	Prerequisites for CAD
The role of the coordinator	
Research and expertise	
Collaboration required	
Mutual goals	Collaboration and Participation
Win-win	
Challenges working together	
CAD Stockholm today	
Formal agreements and policy	Sustainability and dissemination
Keeping up interest	
Importance of long term commitment	

#### Prerequisites for CAD

A needs assessment was conducted in Stockholm to determine whether there was a problem with drugs in the nightclub environment and the severity of drug use. This was emphasized as an essential prerequisite for CAD. The study was used to formulate mutual goals and to gain support from managers and politicians. This support was perceived as substantial.

*Mutual analysis or evaluation, that’s where it starts.* Police officer

The participation of the stakeholders was described as necessary and important; the coordinator who started CAD wanted the stakeholders to feel that it was their project.

*It has been an important part all the time, participation.* Former Coordinator

The coordinator described their work as extensive and exceeding their full-time employment during the first years of CAD. All respondents stressed the importance of coordination, especially when working with multiple stakeholders.

#### Collaboration

Collaboration was described as necessary and well-functioning but the hard work in forming the groundwork to initiate the program was also highlighted. The former coordinator emphasized the importance of their previously positive collaboration with the municipality and the police through STAD and their efforts to reduce alcohol-related problems in the nightclub setting. The difficulties concerning collaboration were described as related to the nature of the stakeholder’s occupations. The club owners were described primarily as businessmen for whom drug use prevention was not the highest priority. Furthermore they were viewed to be in a dependant relationship to the authorities in order to keep their licence to sell alcohol. To develop trust between the authorities and the nightclub industry was described as hard work but also successful overall.

*There are very competent police officers, very competent public officials and also competent club owners, but it is about keeping this gang together. It’s a quite motley crew.* Club owner

#### Sustainability

CAD had been sustained in Stockholm and the current coordinator described the program as being a natural part of community drug preventive work. To achieve sustainability the importance of formal agreements was highlighted which also assured that CAD remained a high priority. Keeping up interest was emphasised as a challenge for sustaining the work.

*I believe in agreements and on being clear about what the police commit to doing.* Police officer

## Discussion

Interest and enthusiasm for CAD was substantial in the communities to which it had been disseminated but at the same time there had been considerable difficulties during the implementation process. There was a discrepancy between the results of the questionnaire and the first interviews. The enthusiasm and contentment expressed in the questionnaires did not, when put in the light of the interviews, correspond with an overall success in implementation. It rather reflected contentment with smaller parts of the program or with the thought of what could be rather than what actually was. Compliance within the program was lacking, the police had had difficulties with their commitments and the work with CAD had ceased almost entirely in two of the three example municipalities. The results differed from the experiences in Stockholm where the work was perceived overall as being successful and sustained. Factors highlighted as important were support, participation and the assessment of needs, among other things. The results are consistent with prior research demonstrating that implementation is complex and that successful implementation requires considerable effort [2]. The capacity to implement and sustain CAD differed between Stockholm and the other municipalities, which will be discussed below. To guide the reader through this comparison, we have summarized the essential differences in implementation factors in Table 5.

**Table 5:** Summary of the comparison of factors related to the implementation of CAD

**Factor**	**Stockholm**	**Other municipalities**
**Perception of needs**	Assessment of needs and perceived these as extensive	Approximately half completed some form of assessment before initiating CAD
**Participation**	Extensive participation in development of the CAD program	No participation in the development phase, small possibilities for adaptation
**Acceptance of program**	Extensive acceptance from stakeholders	Extensive acceptance but not among managers and politicians
**Ability to mobilize resources**	Large communities meant greater opportunity to allocate resources. Resources were perceived as sufficient	Many smaller communities meant less opportunity to allocate resources. Resources within the police perceived as lacking
**Support and engagement from managers and politicians**	Extensive	Lacking, especially higher up within the organizations
**Local dedicated individuals**	Important in the process	Important in the process
**Competence within the community**	Continuous support from researchers. Many stakeholders were highly competent within their respective fields	Varying competence
**Collaboration and relations**	High ambition and capacity to cooperate	High ambition but sometimes lacking capacity

### The assessment needs, participation and acceptance of program

During the 1990s Stockholm experienced a significant rise in problems associated with drugs and criminal activity in the nightclub environment and an investigation was launched to determine the magnitude of illicit drug use in the nightlife setting [1]. These problems were cited by the Stockholm respondents as the primary reason for the initiation of CAD and also for the extensive support, acceptance and legitimacy of the program. In the other municipalities there was no such explicitly expressed need for drug preventive interventions in the club environment and only in about half of the municipalities had some sort of survey of drug problems been made. This is problematic as an implementation should always be preceded by a formal needs assessment, including assessment of the perceived needs of the stakeholders [2,7].

The development of CAD in Stockholm engaged a broad range of stakeholders in the overall process and participants took part in creating the program based on their specific needs and expertise. This stakeholder community empowerment was lacking in the other municipalities to which CAD was disseminated, as they received a fixed program to implement with small possibility for local variations or adaptation.

### The ability to mobilize resources and competence

When the program was developed in Stockholm groundwork had been done for years before, this through collaboration with the clubs on Responsible beverage service (RBS). The organizational prerequisites were in place to facilitate the development and implementation of CAD, this mainly through STAD but also within the police organization. In Stockholm the program had a full-time coordinator who also served as the lead investigator that researched the development, implementation and outcomes of CAD. In the other municipalities the coordinator was a public official that assumed extra duties associated with the implementation of CAD. The coordinator in Stockholm highlighted their full-time focus on CAD as being necessary for success of the project. Considerable time and effort was required by the coordinator to raise interest among various stakeholders, to facilitate stakeholder collaboration and to manage media messages and policy work. In Stockholm there were also additional researchers involved that had a great interest in seeing the success of this project. The external knowledge supporting the other municipalities consisted of the national coordinator who worked 50% with this assignment. Despite the knowledge gained from CAD in Stockholm and the success of the program, technical assistance (TA) was not used in the dissemination of CAD to the rest of the country. The program was designed for use in Stockholm and the developers had no intentions of spreading the program, neither did they partake in the dissemination. Future implementation of similar programs could benefit from the use of TA as this enhances the chances of successful implementation. Currently the CAD program has an annual meeting for participants and this provides the opportunity to make new contacts and share experiences in working with drug problems at the nightclubs. This can, in hindsight, provide a type of technical assistance but more specific efforts to help the dissemination were needed early on.

The requirements and investments of the stakeholders who joined CAD varied. The police was the stakeholder that had to make the biggest commitment and organizational change to manage their part, which included more and closer contact with the clubs and an overall shift in priority with more personnel working towards the clubs. The inability by the police to meet the requirements was, in the municipalities to which CAD had been disseminated, the main reason for why the implementation did not go as desired. Reasons for this inability were said to be mainly scarce resources, reorganizations and lack of interest. In Stockholm the police were engaged in the project from the beginning, they already had efforts towards clubs and saw the potential gains of extending those efforts. The respondents in Stockholm expressed that police involvement was a major reason for positive outcomes of CAD and that they were an indispensable part in the program. Respondents also spoke about the importance of prioritizing drug preventive efforts within the community and the need to have resources specifically allocated to the police for their participation in community-based drug prevention efforts; this may not be possible in smaller communities with limited resources. Overall the stakeholders regarded the police as playing a pivotal role in the CAD program and without their presence found little meaning or incentive in following through with their commitments. In municipalities where the police state that they do not have the capacity to allocate resources for their active participation in CAD or where the support and legitimacy within their organization is lacking, initiation of the program should be questioned. As Stockholm is the largest city in Sweden the possibilities to allocate resources are much greater compared to smaller municipalities. Communities with small populations can have special challenges when it comes to implementing, for example with resource allocation [19]. Broad participation and stakeholder engagement was by respondents stressed as an important tool for drug prevention but in order to work collaboratively the organizational prerequisites have to be adequate in all of the participating organizations [2]. Further the sustainability of the program is dependent on long-term financial and organizational capacity of the stakeholders [3]. The stakeholders are recommended to set up formal agreements to facilitate long-term commitment and to ensure prioritization of the program.

### Legitimacy, collaboration, support and the role of dedicated individuals – necessary circumstances for prioritizing CAD

One central aspect that differed between Stockholm and the majority of the other municipalities was the support from politicians and persons in leading positions. In Stockholm this was a major supporting factor for the implementation. Support was lacking or at least not as extensive in other municipalities. The police had significant problems due to lack of support. Lack of support was described as the main reason for implementation failure. Support from leaders is an essential part in the implementation process and for sustainability and institutionalization of interventions [4,8,20]. It is therefore important that support exists in the implementing communities and that the collaboration between the stakeholders is not dependent on individuals. The persons involved in Stockholm were positioned high up in their organizations and had the legitimacy to pursue the work. In the example municipalities this support was lacking and the implementation was dependent on dedicated individuals, something also mentioned in the questionnaire. There is a problem when the work depends on the dedicated and when it is individuals collaborating and not organizations [12]. The respondents highlighted the problems associated with persons leaving their positions and this leading to a possible termination of initiated actions. This made the work vulnerable. There are potential benefits in separating collaboration projects from the permanent organization as it enables them to develop more freely. But difficulties can arise as a gap is created between the collaborative project and the permanent organizations. There can be difficulties in incorporating projects into the permanent organizations and this jeopardizes sustainability and institutionalization [12]. Without doubt the support of politicians and managers was a vital prerequisite for CAD.

It was evident in the study that collaboration could affect the implementation in both a positive and a negative direction and this was stressed as an important factor by the interviewees. The stakeholders are mutually dependent on each other and have to rely on the others to do their part. Problems with this lead many to think about the need for formal agreements; something also highlighted in research as important and a recommendation for future implementation of CAD. If program adaptation will be done, something virtually impossible to avoid [21], it has to be consistent across the stakeholders and the adaptation made by one has to be accepted by the others.

### Program fidelity

We found that in many municipalities not all components of CAD had been implemented and that the emphasis was placed on drug-training and on media campaigns. This finding is consistent with previous research illustrating that fidelity is often low when programs are applied in real-world settings [8]. The capacity had overall been insufficient to implement essential parts and fully engage a comprehensive systems approach to prevention in the way originally made in Stockholm. Often what was being implemented was not what research had shown to be the most effective activities. This circumstance is not unique and was also evident in a Swedish evaluation studying of alcohol preventive efforts in six municipalities [22]. Therefore it is important to determine and define core components as a guide to what the implementing community should focus on [2]. CAD could benefit from placing emphasis on the parts of the program which are directed towards availability since this is more likely to affect drug use than interventions trying to change the demand [23]. Failed collaboration was an issue and given that the approaches which comprehensively include all aspects of the program are most likely to lead to desired outcomes [24], it is important to focus on the entire work, including all of the stakeholders, rather than just some parts of the intervention. If the capacity to implement the core components or to sustain collaboration and participation from all stakeholders is lacking, the implementation should be questioned.

### Program readiness

One of the reasons for the less successful implementation of CAD in the smaller communities compared to Stockholm is a lower program readiness. Monetary support from the government was made available for the dissemination of CAD throughout Sweden. When it became possible to apply, too little time between the analysis of the Stockholm results and implementation had passed. The applicability of this for other communities had not been checked and not enough time was available to analyze the needs and prerequisites of the respective communities. As governmental support is only available for a short time span, the necessary analyses to estimate program readiness will often be inadequate.

### Strengths and limitations

The respondents in the study expressed views that were highly consistent across stakeholders and communities about what factors had affected the implementation. In the communities where the police participation was perceived as weak the same view was shared by the interviewed police officers. A strength of this study was the broad inclusion of key informants which facilitated a verification and comprehension of the expressed perceptions. Another strength was the inclusion of a variety of municipalities from different areas of Sweden and the fact that the results from these were highly consistent.

There was a low response rate on the questionnaire, but this is consistent with the overall trend in declining response rates in this type of study [25]. This also may be a sign that the CAD program was no longer a priority in some areas. In terms of the interviews, the respondents were individuals highly engaged in the CAD program and thus willing and motivated to participate in the study. This was the case in the three example municipalities and Stockholm. Another limitation was that no doormen were available for interview. The high turnover in this job type meant that those we could reach in 2012 were not employed at the same nightclub during our study period from 2008 to 2010.

This study was originally done as an evaluation of the implementation between 2008 and 2010, it should be noted that some of the included municipalities had initiated the implementation before 2008. However, we assumed that the implementation factors were the same across all municipalities regardless of the date of program initiation and hence the early start date was not a factor in the selection of example municipalities or in the analysis of the questionnaire or interviews.

## Conclusions

The factors influencing the implementation and the difference in implementation outcome between Stockholm and the rest of the country can be summarized by the concept of community capacity. In Stockholm the community had a need, the individual organizations had the resources required as well as the ability to cooperate. This was made possible by the coordinator and external knowledge. In all, this indicated a high capacity within the Stockholm community. In the other municipalities some parts of this may have been high but overall the capacity was lacking. By the concept of capacity one can highlight the complexity of implementation prerequisites and the importance of resources within all participating organizations as well as the ability to perceive needs, formulate goals and to collaborate. All of this is needed to implement and sustain complex programs and solve the problems of substance use.

There has to be capacity within the implementing organizations including assessment of needs, support, sufficient and long-term funding and formal agreements between stakeholders to facilitate the implementation and sustainability of CAD. Implementation of comprehensive programs is complex and if the capacity to implement is not adequately established beforehand the initiation of implementation should be questioned.

It was difficult to find a municipality, besides Stockholm, where CAD had functioned well and was being sustained. In the example municipality that was functioning well according to the questionnaire and where the stakeholders seemed satisfied with the work, there had been no environmental rounds executed and the collaboration with the police was perceived as lacking as they did not have, or did not prioritize, the required resources to attend the clubs as much as wanted. In this instance the level of satisfaction did not relate to proper implementation of program activities. Expressed satisfaction may be based on the shared enthusiasm but this on its own is not sufficient for accomplishing the main goals and objectives of the CAD program.

Just as programs need to be adapted to local context, so does the implementation process. The process may vary between different communities and factors may further have different influence depending on the context and the other factors involved [8]. Hence it is unrealistic to give a manual on how to implement CAD. However, we hope this study will draw attention to important implementation factors to consider; factors that will enhance the chances for program sustainability and desired program outcomes.

The reason for the higher capacity in Stockholm may also be associated with the size of the municipality as well as the ability to mobilize resources and competence. But in this particular case what the respondents highlighted was the perceived need and benefits. There is a need for evaluation and for research programs in locations other than Stockholm and the studied community must have similarities with the communities where the program will be used in the future. The program has to be relevant in all settings and also possible to implement well in all settings.

More studies of the efficacy of CAD are needed and it should not be promoted as an effective method without a sufficient amount of evaluation. The Swedish authorities should take more responsibility for which intervention programs they are spreading and should demand evaluation and closer collaboration with researchers.

## Competing interests

The authors declare that they have no competing interests.

## Authors’ contributions

EN is the main author and has done most of the data collection. She has been active in writing, as have all the other authors. FS has also been project leader, EW has had main responsibility for theories of collaboration and organization, JG has been the foremost collaborator in Stockholm with personal experience of the start in that city. CP, finally, has made the most valuable comments in finalizing the paper. All authors read and approved the final manuscript.

## References

[B1] Gripenberg Abdon J Drug use at licensed premises prevalence and prevention 2012 Karolinska institutet: Stockholm

[B2] Fixsen DL Naoom SF Blase KA Friedman RM Wallace F Implementation research: a synthesis of the literature 2005 Tampa, Florida: University of South Florida, Louis de la Parte Florida Mental Health Institute, The national Implementation Research Network

[B3] Schell SF Luke DA Schooley MW Elliott MB Herbers SH Mueller NB Bunger AC Public health program capacity for sustainability: a new framework Implement Sci 2013 8 15 10.1186/1748-5908-8-15 23375082 PMC3599102

[B4] Wallin E Lindewald B Andreasson S Institutionalization of a community action program targeting licensed premises in Stockholm, Sweden Eval Rev 2004 28 5 396 419 10.1177/0193841X04264951 15358904

[B5] Wiltsey Stirman S Kimberly J Cook N Calloway A Castro F Charns M The sustainability of new programs and innovations: a review of the empirical literature and recommendations for future research Implement Sci 2012 7 17 10.1186/1748-5908-7-17 22417162 PMC3317864

[B6] Wandersman A Florin P Community interventions and effective prevention Am Psychol 2003 58 6–7 441 448 12971190 10.1037/0003-066x.58.6-7.441

[B7] Flaspohler PD Meehan C Maras MA Keller KE Ready, willing, and able: developing a support system to promote implementation of school-based prevention programs Am J Community Psychol 2012 50 3–4 428 444 22618024 10.1007/s10464-012-9520-z

[B8] Rohrbach LA Grana R Sussman S Valente TW Type II translation: transporting prevention interventions from research to real-world settings Eval Health Prof 2006 29 3 302 333 10.1177/0163278706290408 16868340

[B9] Wendel ML Burdine JN RMcLeroy K Alaniz A Norton B Felix MRJ DiClemente RJ, Crosby RS, Kegler MC Community capacity: theory and application Emerging theories in health promotion practice and research 2009 2 San Francisco: Jossey-Bass 277 302

[B10] Butterfoss FD Kegler MC The community coalition action theory Emerging theories in health promotion practice and research 2009 2 San Francisco: Jossey-Bass 237 276

[B11] Graham K Chandler-Coutts M Community action research: who does what to whom and why? Lessons learned from local prevention efforts (international experiences) Subst Use Misuse 2000 35 1–2 87 110 10677877 10.3109/10826080009147688

[B12] Löfström M Inter-organizational collaboration projects in the public sector: a balance between integration and demarcation Int J Health Plann Manag 2009 25 2 136 155 10.1002/hpm.1003 20013878

[B13] Gripenberg Abdon J Wallin E Andréasson S The “clubs against drugs” program in Stockholm, Sweden: Two cross-sectional surveys examining drug use among staff at licensed premises Subst Abuse Treat Prev Policy 2000 6 2 21299852 10.1186/1747-597X-6-2 PMC3045347

[B14] Holder HD Alcohol and the community: a systems aproach to prevention 1998 Cambridge: Cambridge University Press

[B15] Gripenberg J Wallin E Andreasson S Effects of a community-based drug use prevention program targeting licensed premises Subst Use Misuse 2007 42 12–13 1883 1898 18075915 10.1080/10826080701532916

[B16] Abdon JG Wallin E Andréasson S Longterm effects of a communitybased intervention: 5 year followup of Clubs against Drugs Addiction 2011 106 11 1997 2004 10.1111/j.1360-0443.2011.03573.x 21749523

[B17] Doyle L Brady AM Byrne G An overview of mixed methods research J Res Nurs 2009 14 2 175 185 10.1177/1744987108093962

[B18] Graneheim UH Lundman B Qualitative content analysis in nursing research: concepts, procedures and measures to achieve trustworthiness Nurse Educ Today 2004 24 2 105 112 10.1016/j.nedt.2003.10.001 14769454

[B19] Barnidge EK Radvanyi C Duggan K Motton F Wiggs I Baker EA Brownson RC Understanding and addressing barriers to implementation of environmental and policy interventions to support physical activity and healthy eating in rural communities J Rural Health 2012 29 1 97 105 23289660 10.1111/j.1748-0361.2012.00431.x PMC4760835

[B20] Wisdom JP Chor KHB Hoagwood KE Horwitz SM **Innovation adoption: a review of theories and constructs** Adm Policy Ment Health Ment Health Serv Res 2013 Epub DOI 10.1007/s10488-013-0486-4 10.1007/s10488-013-0486-4 PMC3894251 23549911

[B21] Durlak JA DuPre EP Implementation matters: a review of research on the influence of implementation on program outcomes and the factors affecting implementation Am J Community Psychol 2008 41 3–4 327 350 18322790 10.1007/s10464-008-9165-0

[B22] Kvillemo P Andréasson S Bränström R El-Khouri BM Karlsson L Effekter av lokalt alkohol- och narkotikaförebyggaden arbete (Effects of local drug preventive work) 2008 Östersund: Statens folkhälsoinstitut

[B23] Babor T e Drug policy and the public good 2010 New York: Oxford University Press

[B24] Jones L Hughes K Atkinson AM Bellis MA Reducing harm in drinking environments: a systematic review of effective approaches Health Place 2010 17 2 508 518 21257334 10.1016/j.healthplace.2010.12.006

[B25] Baruch Y Response rate in academic studies—a comparative analysis Hum Relat 1999 52 4 421 438

